# Effects of Local Administration of Tranexamic Acid on Reducing Postoperative Blood Loss in Surgeries for Closed, Sanders III–IV Calcaneal Fractures: A Randomized Controlled Study

**DOI:** 10.1007/s43465-021-00417-2

**Published:** 2021-05-22

**Authors:** Lang Zhong, Yu Liu, Yongcai Wang, Hongchuan Wang

**Affiliations:** Department of Orthopaedic Surgery, People’s Hospital of Leshan, Shizhong District, Leshan, Sichuan China

**Keywords:** Calcaneal fracture, Tranexamic acid, Postoperative blood loss, Complication

## Abstract

**Purpose:**

To investigate whether local administration of tranexamic acid (TXA) is effective in postoperative blood loss reduction in surgeries for Sanders III–IV calcaneal fractures.

**Methods:**

Calcaneal fracture patients who were hospitalized in our hospital from August 2014 to April 2018 and underwent open reduction internal fixation (ORIF) via lateral approach with an L-shaped incision were included in the present study. 53 Patients were randomly divided into three groups, groups A (17), B (17) and C (19). Twenty milliliters of 10 mg/ml and 20 mg/ml TXA solution were perfused into the incision of patients in group A and group B, respectively. Twenty milliliters of saline were perfused into the incision of patients in group C as control. The volume of postoperative drainage, postoperative blood test, coagulation test, and wound complications were analyzed to evaluate the effectiveness of local administration of TXA on blood loss reduction.

**Results:**

The amount of drainage at 24 and 48 h after the procedure was 110 ± 170, 30 ± 10 ml and 130 ± 160, 20 ± 17 ml for patients in group A and group B, respectively. The corresponding numbers for patients in group C were 360 ± 320, 20 ± 10 ml. The difference between group A and group C was statistically significant, so was the difference between group B and group C. No statistically significant difference was found between group A and group B. Postoperative blood test results revealed that the levels of hemoglobin and hematocrit were significantly higher in group A and group B when each compared to that of group C. In contrast, no difference was found between group A and group B. No significant difference was found between each experimental group and the control group in terms of platelet counts, prothrombin time (P.T.), activated partial prothrombin time (APTT), and wound complications.

**Conclusion:**

Local administration of TXA is effective in the reduction of postoperative blood loss in surgeries for Sanders III–IV types of calcaneal fractures without notably associated side effects.

## Introduction

Calcaneal fractures account for approximately 2% of all bone fractures and represent 60% of fractures of tarsal bones [1]. For closed, Sanders III–IV calcaneal fractures, open reduction internal fixation (ORIF) is the standard treatment. However, a large amount of perioperative blood loss is usually associated with the surgeries to repair calcaneal fractures as calcaneus is a cancellous bone with abundant blood supply, which increases the chance of blood transfusion and the associated risks to patients with calcaneal fractures [2]. The usage of tourniquet in calcaneal fracture repairing surgeries significantly reduced the blood loss during the procedure, but the amount of postoperative blood loss remains large. Moreover, the large amount of postoperative blood loss around the affected calcaneus will also increase the risk of wound complications [3]. Therefore, new approaches to reduce postoperative blood loss in calcaneal fracture repairing surgeries remain to be developed [4].

Tranexamic acid (TXA) is a synthetic analog of lysine that functions as an antifibrinolytic agent through the competitive binding to the lysine sites on plasminogen, plasmin, and plasminogen activator [5], by which it inhibits fibrinolysis and thrombolysis to reduce the blood loss due to the surgeries effectively. TXA is widely used in cardiovascular surgeries, hip and knee surgeries, and spine surgeries [6–8]. Recently, more and more clinicians are interested in the local administration of TXA in surgeries due to the risk of deep venous thrombosis induced by intravenous usage of TXA [9]. Kim and the colleagues reported that TXA was more effective when used intraarticularly than intravenously with respect to the reduction of perioperative blood loss and lowering the blood transfusion rate [10]. In the present study, we investigated the effects of local administration of TXA in the incision on blood loss reduction in the ORIF procedure for calcaneal fractures. Postoperative drainage volume wound complications, postoperative coagulation index, and other factors were analyzed to evaluate the effects of local administration of TXA on the reduction of postoperative blood loss in surgeries for closed, Sander III–IV calcaneal fractures.

## Patients and Methods

### Ethics Approval and Consent to Participate

This study was approved by the local institutional review board of Leshan People's Hospital. Written informed consent (including patients' details, images, or videos) was obtained from all participants. All experiments were performed in accordance with relevant guidelines and regulations. This study was conducted in accordance with the Declaration of Helsinki.

Patients hospitalized in Leshan People's Hospital from August 2014 to April 2018 for ORIF to repair calcaneal fractures were included in the present study, including 42 males and 11 females. Patients were randomly divided into three groups, with a random number table method. There were 17, 17, and 19 patients in experimental group A, experimental group B, and control group C, respectively.

### Inclusion Criteria

The inclusion criteria were as the following: (1) patients who underwent ORIF with conventional plates and pins via extended lateral approach with L-shaped incision for closed, Sanders III–IV calcaneal fractures; (2) patients' age ranged from 18 to 70 years; and (3) patients were fully informed about the surgeries and the trial, and the consent form was signed by each participated patient. The trial was approved by the ethics committee of the hospital.

### Exclusion Criteria

Patients were excluded from the present study if met one or more of the following criteria: patients who had coagulation problem before the procedure (preoperative platelet count < 1.5 × 10^5^/mm^3^, international normalized ratio (INR) > 1.4, or activated partial thromboplastin time (APTT) > 1.4-folds of normal range); patients with impaired liver function or kidney function; patients who had peripheral vascular diseases, history of vascular thrombosis, or history of long-term usage of anticoagulation medications.

### TXA Solution Preparation

**Fig. 1:**
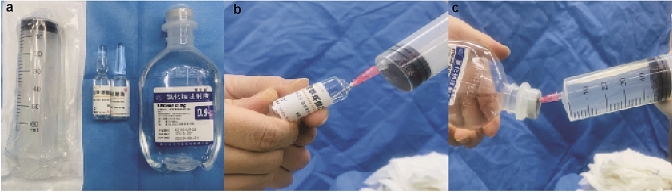
TXA solution preparation. **a** Tools and drugs used in the preparation of TXA solution. Left: 60 ml syringe; middle: TXA stock solution in vial (100 mg/ml); right: physiological saline used. **b** Drawing TXA stock solution into the syringe from the vial. **c** Drawing physiological saline into the same syringe containing desired amount of TXA stock solution

TXA solution was prepared in a syringe under sterile condition. 5 ml TXA stock solution (100 mg/ml) (Tranexamic Acid Injection®, TIANXIN CO.LTD, Guangzhou, China) was first drawn into a 60 ml sterile syringe, then 45 ml of physiological saline was drawn into the same syringe to dilute the TXA to 10 mg/ml. The solution was mixed well by gently shaking. The 20 mg/ml TXA solution was prepared in similar way except diluting the 5 ml TXA stock solution with 20 ml physiological saline. The syringe and the procedure are shown in Fig. 1 with representative photos.

### Surgical Procedures

**Fig. 2:**
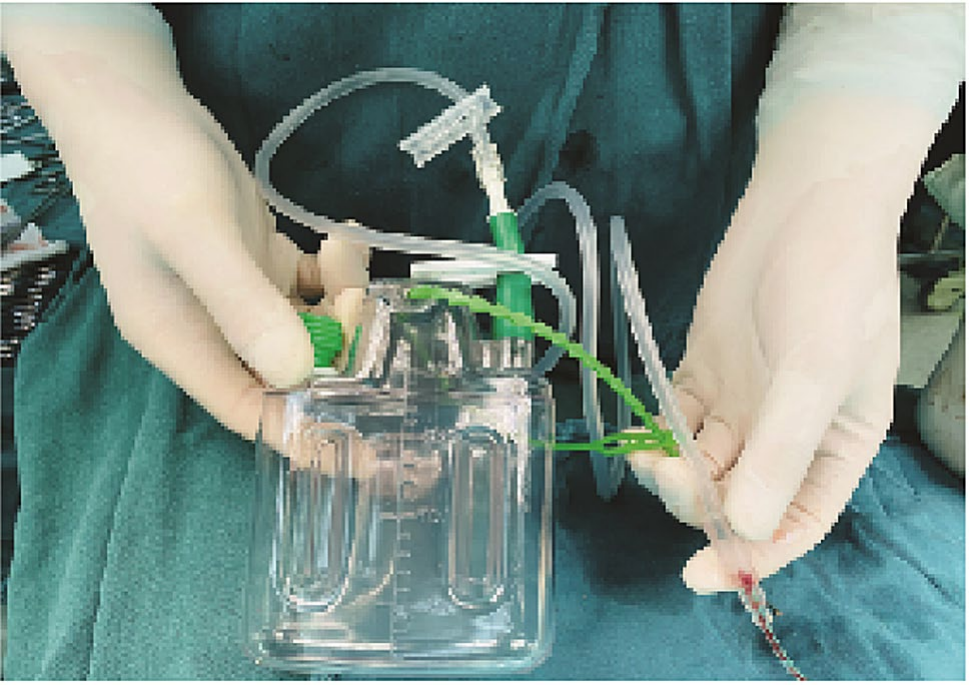
Representative photo showing the drainage tube connected to a 200 ml drainage bottle with negative pressure

**Fig. 3:**
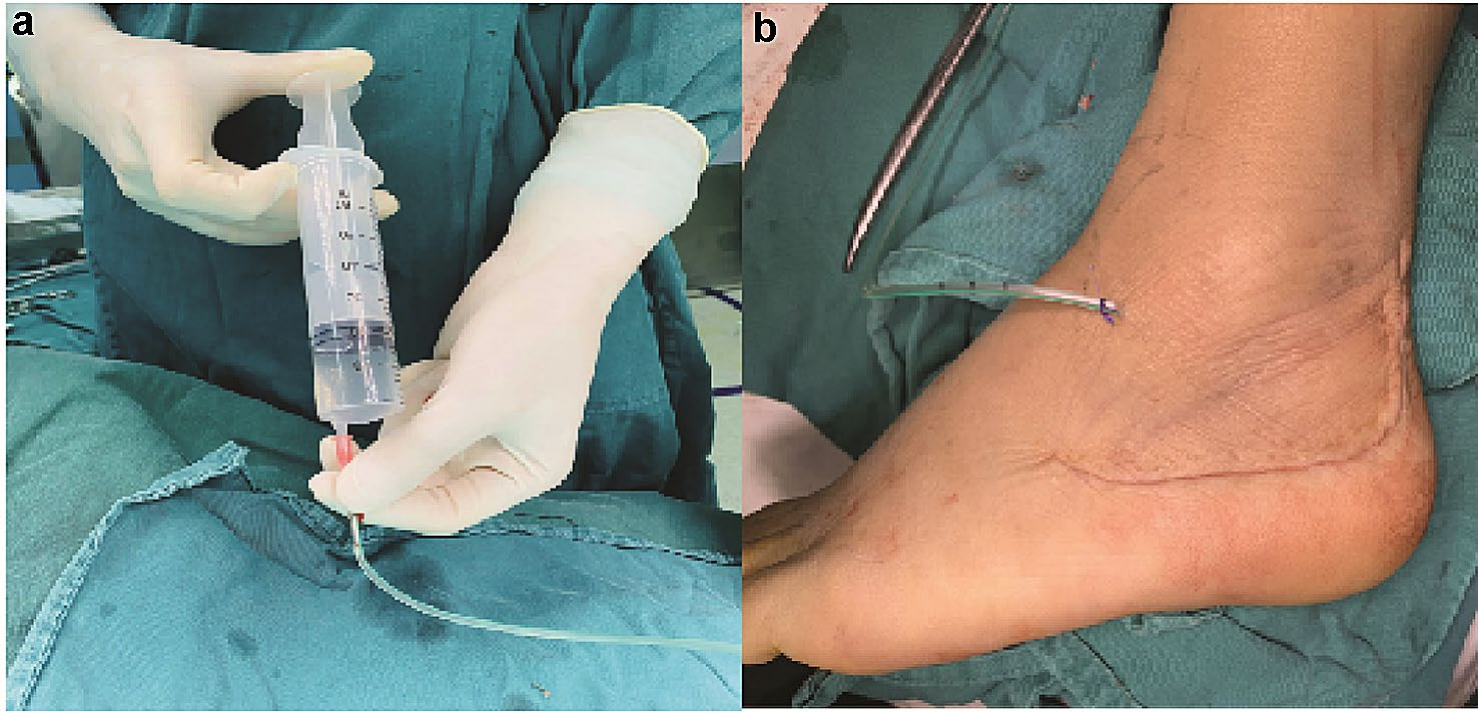
Drainage tube placement and wound closure. **a** Representative photo showing perfusing TXA solution into the wound. **b** Representative photo showing the Closed wound with a drainage tube being placed

Surgical procedures were performed when the affected foot showed no signs of swelling and/or blisters (dermatoglyphic pattern positive). All the surgeries were performed by the same surgeon. Patients laid down on the healthy side, and tourniquets were used on the affected limb. The pressure for the tourniquet was set as 100 mmHg higher than the patient's arterial pressure. An L-shaped incision was made dorsolateral to the fractured calcaneus to achieve a sharp, subperiosteal dissection for a full-thickness flap. The L-shaped incision was made up to the subtalar joint level vertically and stopped at the calcaneocuboid joint horizontally. Once the incision was made, the full-thickness flap was held in place with three 2.0 mm Kirschner wires by screwing one Kirschner wire into each of fibula, talus and cuboid tightly close to, and underneath the full-thickness flap. Then the incision was opened by static traction to expose the calcaneocuboid joint and subtalar joint. Bohler angle and Gissane angle were restored by prying. The length, height, and width of the calcaneus were restored using c-arm pliers. Allograft bone was used to fill the large defect that remained after the reduction. Once the reduction was satisfactorily performed, fixation was performed with the calcaneal plate. Before removing the tourniquet, the drainage tube (external 6 mm, internal 3 mm) was placed into the incision, and the incision was closed. 20 ml TXA solution or saline were perfused into the incision through the drainage tube. Then the drainage tube was clamped and connected to a 200 ml negative pressure drainage bottle. A representative picture of the drainage tube connected to a negative pressure drainage bottle is shown in Fig. 2. In addition, a representative figure demonstrating the position of drainage tube placement in the closed wound was shown in Fig. 3.

### TXA Treatment and Postoperative Management

20 ml TXA solution was perfused into the incision of patients. The concentration of TXA in the solution was 10 mg/ml and 20 mg/ml for group A and group B, respectively. Plain saline without TXA was used as a control to be perfused in the control group. The incision was bandaged with compression for patients in all groups. The drainage tube was re-opened 2 h after the clamping of the drainage tube, and it was removed 48 h after the surgery. Routine management of patients included the application of antibiotics for 24 h. The affected foot was intermittently iced and raised for 3 to 4 days. Patients started extension and flexion training for the affected ankle joint 2 weeks after the surgery and started weight training for the ankle 3 months after the surgery.

### Outcome Measurement

Baseline clinical characteristics were monitored to assess the homogeneity among the groups. Collected data included age, gender, BMI index (Body mass index), preoperative blood test, anesthesia method, types of fracture, and the surgery duration.

The primary outcome measurements included the volume of drainage 24 h and 48 h after the procedure. The volume of postoperative drainage was calculated by subtracting 20 ml (the amount of perfused solution) from the total volume of drainage at the indicated time. Other primary outcomes monitored included postoperative routine blood test and coagulation test, as well as wound complications, including dehiscence, peri-wound necrosis, infection, and hematoma.

### Statistical Analysis

SPSS 17.0 software was used to analyze the data. Data were presented as mean ± SD. One-way ANOVA was used to analyze categorical data between two groups, and *p* < 0.05 was considered significant. Two-sided Chi-squared test was used to analyze numerical data, and α value was set to 0.05.

## Results

Overall, 53 patients with Sanders III–IV calcaneal fractures participated in the present study, including 42 males and 11 females. They were randomly divided into three groups, group A, group B and group C. 20 ml of 10 mg/ml and 20 mg/ml TXA solution was perfused into the closed incision via drainage tube for patients in group A and group B, respectively. Moreover, 20 ml of physiological saline was perfused in the closed incision for patients in group C as control. The preoperative baseline data and the postoperative outcomes were analyzed to evaluate the effects of TXA local administration in surgeries for calcaneal fractures.

### Preoperative Baseline Data

As illustrated in Table 1, No significant difference was found among the three groups in terms of preoperative baseline data, including gender, age, BMI index, preexisting conditions (such as diabetes, hypertension, hypothyroid etc.), waiting time until the operation, and the preoperative routine blood test results.

**Table 1:** Baseline data of participated patients

	Group A	Group B	Group C	*p* value
Gender (M/F)	13/4	14/3	15/4	1.000
Age (years)	43.1 ± 8.5	40.4 ± 9.0	40.4 ± 8.9	0.573
BMI (kg/m^2^)	23.8 ± 2.6	23.8 ± 2.6	24.0 ± 2.5	0.965
Pre-existing conditions (Yes/No)	3/14	3/14	4/15	1.000
Waiting period before surgery	11.5 ± 2.3	11.8 ± 2.7	11.8 ± 2.7	0.931
Preoperative blood test				
Hemoglobin (g/dl)	12.2 ± 0.85	12.3 ± 1.47	12.2 ± 1.24	0.960
Platelet count (109/l)	255.0 ± 38.0	257.5 ± 40.6	256.4 ± 28.8	0.979
Hematocrit (%)	38.8 ± 3.6	38.7 ± 4.3	39.2 ± 3.3	0.309
PT (S)	12.5 ± 1.3	13.1 ± 0.9	12.7 ± 1.4	0.788
APTT (S)	38.1 ± 4.6	38.6 ± 3.9	38.3 ± 3.7	0.595

BMI: Body mass index; APTT: activated partial thromboplastin time, P.T.: prothrombin time; Numbers were presented as mean ± SD

### Primary Outcomes

The most important primary outcome we would like to analyze in the present study was the postoperative blood loss. As shown in Table 2, the average amount of postoperative drainage amount at 24 and 48 h were 110 ± 170、30 ± 10 ml for patients in group A, and 130 ± 160, 20 ± 17 ml for patients in group B, respectively. Those numbers mentioned above were significantly less compared to the corresponding numbers of patients in group C (control group), which were 360 ± 320, 20 ± 10 ml, respectively (*p* < 0.01; *p* < 0.05, respectively). No statistically significant difference was found between group A and group B with respect to the drainage volume, indicating that the concentration of 10 mg/ml of TXA is high enough to effectively reduce the postoperative blood loss (*p* = 0.664; *p* = 0.844, respectively). Consistently, a postoperative routine blood test revealed that the average levels of hemoglobin and hematocrit were significantly higher in group A and group B compared to those in group C (*p* = 0.008, *p* = 0.000, respectively). However, no difference was found between group A and group C, nor between group B and group C in terms of platelet count, prothrombin time (P.T.), and activated partial thromboplastin time (APTT), suggesting that the systematic coagulation was not affected by the local administration of TXA.

**Table 2:** Postoperative blood loss and blood test result

	Group A	Group B	Group C	*p* value
Postoperative drainage (ml)				
24 h	110 ± 170	130 ± 160	360 ± 320	0.000*
48 h	30 ± 10	20 ± 17	20 ± 10	0.022*
Postoperative blood test				
Hemoglobin (g/dl)	12.3 ± 0.9	12.2 ± 1.1	10.8 ± 1.6	0.008*
Platelet count (109/l)	245.0 ± 56.2	245.0 ± 56.2	245.0 ± 56.2	0.869
Hematocrit (%)	38.1 ± 3.5	37.8 ± 3.2	32.2 ± 3.6	0.000*
PT (S)	12.7 ± 1.4	13.1 ± 1.3	12.3 ± 1.1	0.178
APTT (S)	37.6 ± 4.4	38.2 ± 3.8	38.5 ± 3.6	0.595

APTT: activated partial thromboplastin time; P.T.: prothrombin time, numbers were presented as mean ± SD. *p < 0.05

**Fig. 4:**
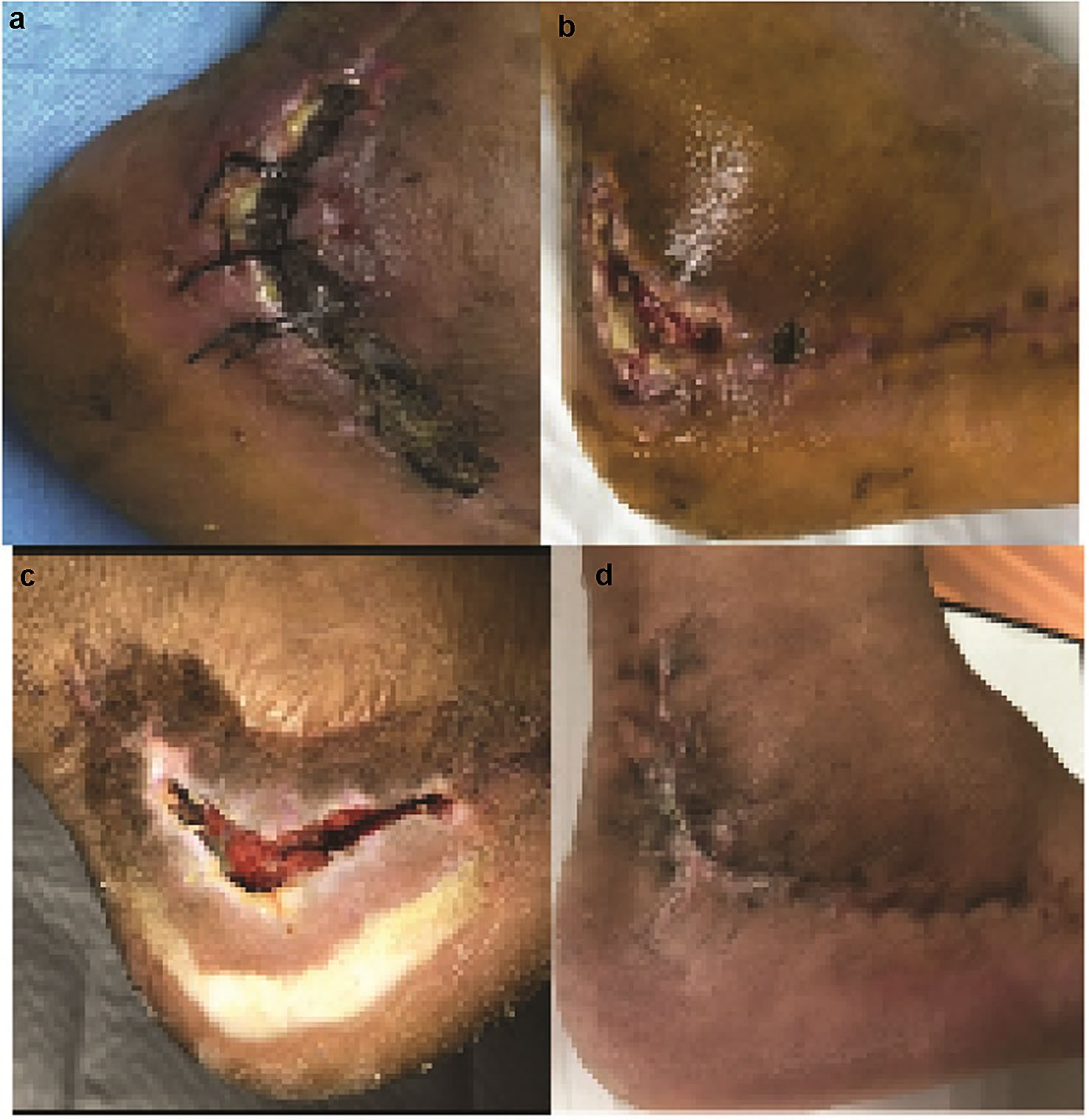
Representative photo of wound complications. **a**, **b**, **c** Wound; **d** healed wound

We also investigated the effect of TXA administration on the occurrence of common wound complications, including dehiscence, peri-wound necrosis, infection, and hematoma. No significant difference was found between the experimental groups and the control group. The detailed result is listed in Table 3. No systemic complication such as deep vein thrombosis was found in the study. Patients who had wound complications in all groups received corresponding treatments according to their specific complications. For those patients who had periwound necrosis and/or superficial infection, a prophylactic empirical course of oral antibiotics and wound care with damp-to-dry dressing changes were applied until the wound healed. Figure 4 shows a representative process of wound healing under the treatments in a patient in group C.

**Table 3:** Postoperative wound complications

Types of complication	Group A	Group B	Group C	*p* value
Dehiscence	0	0	0	1.000
Periwound necrosis	2	3	1	0.503
Hematoma	0	0	1	1.000
Superficial infection	1	0	2	1.000
Deep infection	0	0	0	–
Total	3	3	4	0.955

## Discussion

Although tourniquets can be used to reduce the intraoperative blood loss in surgeries to repair the calcaneal fracture, the amount of postoperative blood loss is still relatively large. A large amount of postoperative blood loss will result in the deterioration of patients' overall health status and will increase the chance of transfusion for patients, especially for those who have a compound injury or those who have severe, chronic underlying conditions [9]. Moreover, using tourniquets for a long time will not only lead to ischemia and hypoxia but also cause ischemia–reperfusion damages to the affected limb after removing the tourniquet, which will result in the activation of tissue plasminogen and ultimately the increase of postoperative blood loss. This is particularly true within 6 h after the tourniquet removal [10].

TXA, a synthetic analog of lysine, can be used to inhibit the activities of core enzymes in fibrinolysis, such as plasminogen and plasmin. It is reported that TXA could reduce perioperative blood loss by inhibiting fibrinolysis [11]. TXA has been widely used in various types of surgeries, and the dosage and using conditions have been discussed. Xie et al. reported in a randomized controlled trial that intravenous injection of 15 mg/ml TXA solution before making the incision significantly reduced the drainage volume in surgeries for Sanders II–III types of calcaneal fractures [12, 13].

However, one of the risks of systematic administration of TXA is to increase the chance of deep vein thrombosis after hyperfibrinolysis caused by TXA, which is also the case for TXA administration in surgeries [14]. Therefore, researchers investigated the possibility of local administration of TXA in surgeries. Ma et al. described the local administration of TXA in the procedure of total knee arthroplasty by perfusing TXA solution into the closed incision via drainage tube and clamping the tube for a certain amount of time to maintain the local treatment of the affected tissue with TXA [15]. It turned out that the local administration of TXA significantly reduced postoperative blood loss without increasing the occurrence of complications. Based on these findings, we tested the effects of local administration of TXA in surgeries for the calcaneal fracture to see if it can reduce the blood loss without inducing other complications. The results presented in our study demonstrated perfusion of TXA into the incision effectively reduced the postoperative drainage amount at day one and day two after the surgery without increasing the risk of coagulation, and the postoperative hemoglobin level was higher in TXA treated patients than that of saline-treated patients, suggesting that local administration of TXA is a promising approach to avoid large amount postoperative blood loss in surgeries for calcaneal fractures. Notably, we did not find the higher concentration of TXA (20 mg/ml) was better than the lower concentration of TXA (10 mg/ml) in terms of reducing blood loss, suggesting that the concentration of 10 mg/ml of TXA is enough.

Further investigations are required to optimize the dosage of TXA and the Duration of treatment to get better results. Xie and his colleagues reported a similar effect of TXA on the blood loss reduction in calcaneal fracture surgeries [13]. In their study, they found less blood loss after surgery and no significant increase of thromboembolic events or adverse drug reaction in TXA treatment group compared to that of control group. Compared to their study, we treated the wound locally, resulting in a hugely reduced amount of TXA application. Based on the dramatically reduced amount of TXA usage, we speculated that TXA treatment in our study may reduce the chance for systematic complications caused by TXA, such as thrombosis, even more. Furthermore, local administration, instead of intravenous injection of TXA may also make the risks of systematic complications induced by TXA lower. More studies are required to confirm the speculation.

In addition to the blood loss, we also investigated whether local treatment by TXA has an effect on the occurrence of postoperative complications. Although the result demonstrated no difference between TXA treated groups and the control group, the greatly reduced postoperative blood loss may reduce the risk of complications. Moreover, as an anti-fibrinolysis agent, TXA inhibits the activity of plasmin, leading to the inhibition of pro-inflammatory factors, such as monocytes, neutrophils, platelet, endothelial cells, complement system and cytokines, which may reduce the risk of wound complications such as infection [12, 13]. More cases need to be studied to reach a conclusion.

To our knowledge, this is the first report about the local administration of TXA in calcaneal fracture repairing surgeries. The participated patients in each group showed no difference in terms of baseline data, including age, gender, waiting time before surgery, underlying medical conditions, and preoperative coagulation. Furthermore, all the surgeries were performed by the same experienced surgeon with the same approach, making the data from each group comparable to the biggest extent. However, the size of the trial in our study is relatively small; secondly, the present study excluded patients having underlying conditions such as abnormal coagulation or history of thrombosis, in whom local administration of TXA might have greater benefits on the reduction of blood loss and postoperative complications. A larger sized trial, including those patients having various preexisting conditions, is required to comprehensively evaluate the benefits of local administration of TXA, especially surgery-associated complications.

## Conclusions

In the present study, we validated that local administration of TXA effectively reduced the postoperative blood loss in surgeries for Sanders III–IV types of calcaneal fractures without increasing the incidence of wound complications, suggesting the potential benefits of local administration of TXA in treatment for calcaneal fractures.
